# Network Analyses Predict Small RNAs That Might Modulate Gene Expression in the Testis and Epididymis of *Bos indicus* Bulls

**DOI:** 10.3389/fgene.2021.610116

**Published:** 2021-04-30

**Authors:** Andressa O. de Lima, Juliana Afonso, Janette Edson, Esteban Marcellin, Robin Palfreyman, Laercio R. Porto-Neto, Antonio Reverter, Marina R. S. Fortes

**Affiliations:** ^1^Department of Production and Animal Health, School of Veterinary Medicine, São Paulo State University (UNESP), Araçatuba, Brazil; ^2^Department of Animal Science, University of São Paulo/ESALQ, Piracicaba, Brazil; ^3^School of Chemistry and Molecular Biosciences, The University of Queensland, St Lucia, QLD, Australia; ^4^Australian Institute for Bioengineering and Nanotechnology (AIBN), The University of Queensland, St Lucia, QLD, Australia; ^5^CSIRO Agriculture and Food, Queensland Bioscience Precinct, St. Lucia, QLD, Australia

**Keywords:** bovine, RNA-sequencing, systems biology, spermatozoa, miRNA, bta-mir-2886, small RNAs, spermatogenesis

## Abstract

Spermatogenesis relies on complex molecular mechanisms, essential for the genesis and differentiation of the male gamete. Germ cell differentiation starts at the testicular parenchyma and finishes in the epididymis, which has three main regions: head, body, and tail. RNA-sequencing data of the testicular parenchyma (TP), head epididymis (HE), and tail epididymis (TE) from four bulls (three biopsies per bull: 12 samples) were subjected to differential expression analyses, functional enrichment analyses, and co-expression analyses. The aim was to investigate the co-expression and infer possible regulatory roles for transcripts involved in the spermatogenesis of *Bos indicus* bulls. Across the three pairwise comparisons, 3,826 differentially expressed (DE) transcripts were identified, of which 384 are small RNAs. Functional enrichment analysis pointed to gene ontology (GO) terms related to ion channel activity, detoxification of copper, neuroactive receptors, and spermatogenesis. Using the regulatory impact factor (RIF) algorithm, we detected 70 DE small RNAs likely to regulate the DE transcripts considering all pairwise comparisons among tissues. The pattern of small RNA co-expression suggested that these elements are involved in spermatogenesis regulation. The 3,826 DE transcripts (mRNAs and small RNAs) were further subjected to co-expression analyses using the partial correlation and information theory (PCIT) algorithm for network prediction. Significant correlations underpinned the co-expression network, which had 2,216 transcripts connected by 158,807 predicted interactions. The larger network cluster was enriched for male gamete generation and had 15 miRNAs with significant RIF. The miRNA bta-mir-2886 showed the highest number of connections (601) and was predicted to down-regulate *ELOVL3*, *FEZF2*, and *HOXA13* (negative co-expression correlations and confirmed with TargetScan). In short, we suggest that bta-mir-2886 and other small RNAs might modulate gene expression in the testis and epididymis, in *Bos indicus* cattle.

## Introduction

Spermatozoid is the most specialized cell in mammalian organisms. Spermatogenesis, the differentiation of male germ cells, relies on a complex network of specialized molecular mechanisms that are critical to male fertility (MacLean and Wilkinson, 2005; Marengo, 2008; Hermann et al., 2018). During spermatogenesis, three sequential phases of cell proliferation and differentiation occur, where there is an extensive multiplication and proliferation of spermatogonial stem cells, followed by a meiotic division, and finally a remodeling of the nuclear and cellular components forming sperm cells (Abou-Haila and Tulsiani, 2000). Spermatogenesis starts with the multiplication of spermatogonial stem cells followed by their meiotic division into spermatids, which then differentiate into spermatozoa that are released into the lumen of seminiferous tubules in the testis (Staub and Johnson, 2018). Spermatozoa leaving the testis transit through the epididymis, where they further mature, acquiring motility and the ability to fertilize the egg (Cornwall, 2009). The epididymis is composed of *caput* (head), *corpus* (body), and *cauda* (tail), consisting of region-specific characteristics, including a region-specific luminal protein profile (Cornwall, 2009). The spermatozoa from the testis pass to the epididymis, which contributes to their maturation (Belleannée et al., 2012). In the epididymis, secreted luminal proteins, water, and solute balance contribute to the luminal environment necessary for sperm maturation (Huang et al., 2006). Fully formed mature sperm cells emerge from the tail epididymis and are stored until the ejaculation event in the *vas deferens*.

Recently, Hombach and Kretz (2016) have proposed a role for small RNAs in the testis and epididymis: they may be key regulators of gene expression in spermatogenesis, as they are in most cellular processes. RNA polymerase II transcribes small RNAs, and because of this, their expression is mostly regulated by mechanisms that regulate RNA polymerase II activity, such as the interaction of transcription factors and specific DNA sequences (Fuda et al., 2009). Some classes of small RNAs, such as micro (miRNAs), small nuclear (snRNAs), and small nucleolar (snoRNAs) RNAs, play a role in spermatogenesis by being involved in meiosis (Pradillo and Santos, 2018). Small RNAs regulate sperm maturation through mRNA-silencing mechanisms (Nixon et al., 2019), such as destabilizing mRNAs via deadenylation complexes (Bartel, 2018). In addition, miRNAs are important to maintain the epididymis homeostasis and function (Nixon et al., 2019). Small RNAs are present in epididymosomes (Sullivan, 2016) and can modulate mRNA expression in spermatozoa during the epididymal transit (Belleannée, 2015).

Considering the different roles played by the testis and epididymis, some studies investigate the pattern of gene expression of male tract reproductive tissues to shed light on the biological processes related to each specific tissue. Among these studies, there was a characterization of epididymis gene expression in humans (Thimon et al., 2007; Browne et al., 2015) and yak (Zhao et al., 2019). Guyonnet et al. (2009) have reported the differences in the expression pattern between the testis and epididymis in boar. However, knowledge of gene expression patterns in the testis or epididymis of *Bos indicus* bulls is lacking, and the hypothesized role of small RNAs in these tissues remains to be confirmed.

By sampling biopsies from testicular parenchyma (TP), head epididymis (HE), and tail epididymis (TE), we obtained different cell groups that are representative of spermatogenesis in three different stages. In the TP, Sertoli, Leydig, and differentiating male germ cells represent a group of cells with the DNA still bound to histones. In TE and HE, sperm cells are further along their differentiation process, and protamines instead of histones are observed, which is typical of mature sperm cells as described before (Fortes et al., 2014). Therefore, when sampling these tissues, we opened a window to investigate spermatogenesis. Our aim was to combine RNA sequencing, differential gene expression, functional enrichment, and co-expression analyses to investigate potential transcript interactions in the male reproductive system, using *Bos indicus* bulls as a model organism.

Studies on the testicular transcriptome, such as this one, are not only useful for understanding male fertility but also very helpful for genome annotation. Testicular tissue may be under less evolutionary pressure and this can be promoting duplication of protein-coding events and an overabundance of non-coding RNAs (ncRNAs), and not all the protein-coding genes expressed are functional (Soumillon et al., 2013). It is generally reported that the testes have higher gene expression than other tissues (Soumillon et al., 2013; Uhlén et al., 2015). The data reported on this study is available through the Functional Annotation of Animal Genomes (FAANG) Consortium for further research^1^.

## Materials and Methods

### Samples and Data

All the experimental procedures were conducted and approved by the ethics committee of the University of Queensland, Brisbane, Australia (protocol number: ANRFA/SCMB/094/16). Tissue samples were collected after euthanasia of cattle for commercial purposes, as part of normal beef industry activities. Testicular samples (*n* = 4) from mature Brahman bulls (approx. 2 years old) were collected shortly after slaughter and delivered to the research team, who performed the biopsies. For each bull, we performed three biopsies: testicular parenchyma (TP), head epididymis (HE), and tail epididymis (TE). Each biopsy (approximately 50 mg of tissue) was collected in Eppendorf tubes with 1 ml of RNA*later*^®^ (RNA stabilizing reagent, Ambion Inc., Austin, TX, United States). The biopsies were left to stabilize in a cold room overnight. After that, the RNA*later*^®^ fluid was pipetted out, and the tubes with tissue samples were stored in a −80°C freezer until RNA extraction.

### RNA Extraction and Integrity

Biopsy samples were homogenized with Precellys 25 system with zirconium oxide beads (Bertin Technologies SAS, Montigny-le-Bretonneux, France). Following homogenization, RNA was extracted using the total RNA extraction protocol, with the RNeasy kit (QIAGEN Pty Ltd., Melbourne, VIC, Australia). After DNAse treatment, using TURBO DNAse I, each sample was purified using the Zymo Clean and Concentrator Kit as per the manufacturer’s instructions (Zymo Research, CA, United States). The RNA concentration was measured by a NanoDrop ND-1000 spectrophotometer (Thermo Fisher Scientific, Wilmington, DE, United States). Samples without the optimal 260:280 ratio, which was between 1.8 and 2.1, were excluded from the experiment. The RNA integrity was verified by Agilent Bioanalyzer (Agilent, Santa Clara, CA, United States), and only samples with an RNA integrity number (RIN) above eight (RIN > 8) were used for RNA sequencing. When needed, RNA extraction was repeated to achieve this quality and integrity.

### RNA Sequencing, Data Processing, and Quantification

Library preparation and RNA sequencing were performed following the standard Illumina protocols for the HiSeq platform (Illumina, San Diego, CA, United States). The library prep kit was the Illumina stranded total RNA kit with Ribo-Zero Gold (Illumina, San Diego, CA, United States). Pair-end 125-base pair (bp) sequencing was conducted across three lanes of an Illumina HiSeq 2000 v4 analyzer (Illumina Inc., San Diego, CA, United States) using standard protocols, generating approximately 60 to 100 million reads per sample. All the samples were run across all the lanes used, in order to avoid any lane effect on our dataset. The quality control procedure included removing adaptors and short reads. The software TrimGalore 0.4.5 was used for trimming adaptors and for the removal of short reads, where one of the pair-end reads was shorter than 20 base pairs^2^. Before trimming, all reads were 126-bases long, and after trimming, lengths ranged from 20 to 126. Trimming was run in paired mode to avoid unpaired reads after trimming. The quality of trimmed reads was high as evaluated with FastQC 0.11.7^3^, and no quality cut off was required.

The sequencing reads were aligned to the *Bos taurus* genome assembly (UMD 3.1 assembly available in Ensembl database) using the HISAT2 v.2.1.0 (Kim et al., 2015) following the mapping evaluation by Qualimap 2.2.1 (Okonechnikov et al., 2016), reporting only known transcripts from the current bovine annotation. The “reads per kilobase per million mapped reads” (RPKM = total exon reads/mapped reads in millions × exon length in kilobase) were calculated and log2 transformed for data normalization (Mortazavi et al., 2008). To further normalize the gene expression values, we used a mixed model approach that considered the effects of library, tissue, and gene-by-tissue interaction as previously detailed (Reverter et al., 2005; Cánovas et al., 2014). In brief, the mixed model contained the sequencing library treated as a fixed effect, while the interaction of tissue, gene, and animal were fitted as random effects. Fitting this animal, gene and tissue interaction is a robust methodology, commonly used in gene expression experiments to reduce the noise. We were able to fit tissue as we had three different tissues per animal: TP, HE, and TE. The VCE6 software^4^ was used to solve the mixed model equations and to estimate variance components associated with random effects. The normalized gene expression values were used in all subsequent analyses, including differential gene expression.

### Differential Expression Analysis

To identify differentially expressed (DE) transcripts (protein-coding and small non-coding RNAs) in specific regions of the epididymis (head and tail) and in the testis (testicular parenchyma), we carried out pairwise comparisons among the epididymis (head and tail) and testis (testicular parenchyma) tissues.

**FIGURE 1:**
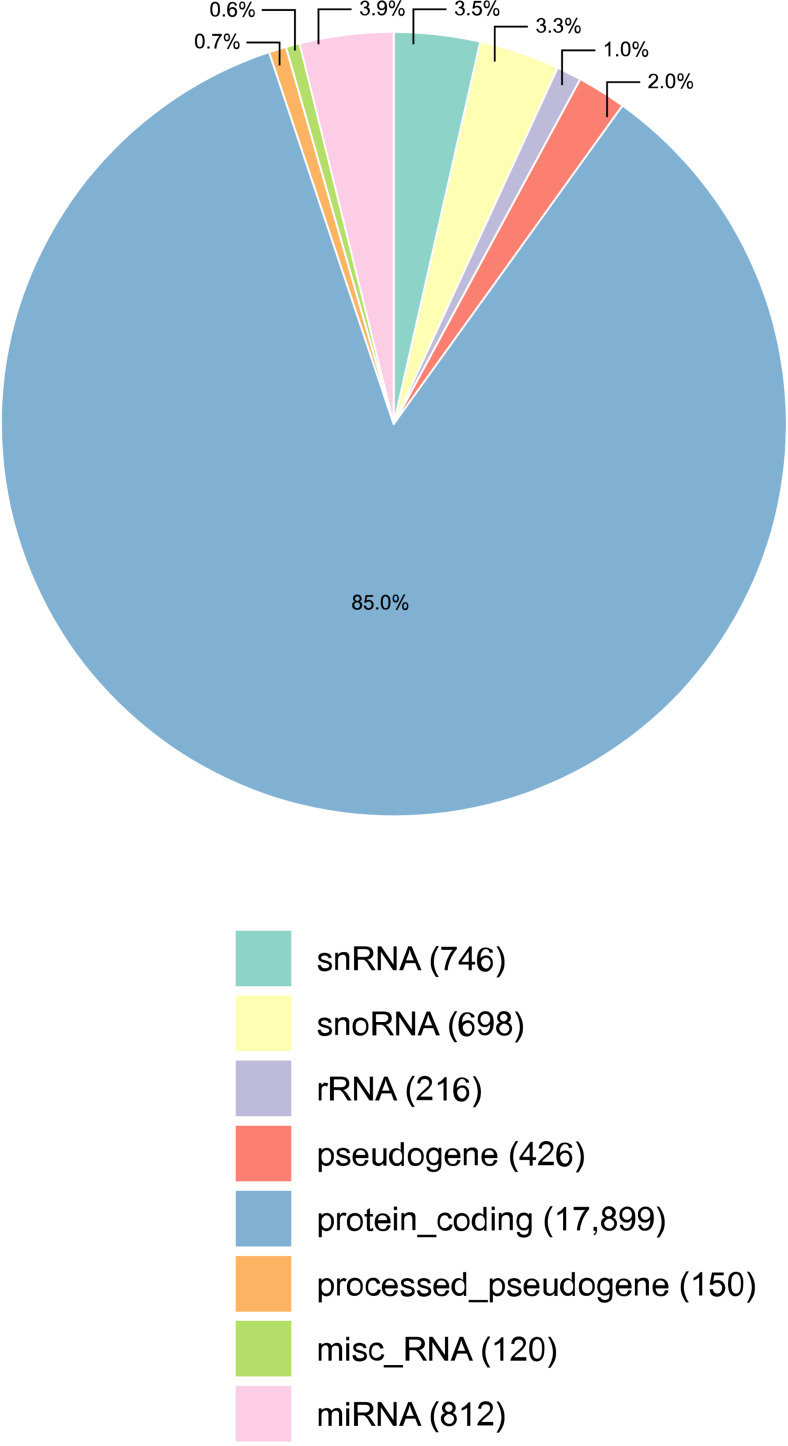
Percentage of transcripts detected per annotation category, in all samples of the male reproductive tract (epididymis and testis) collected from four *Bos indicus* bulls. The majority of the transcripts detected were protein coding mRNAs. The absolute number of transcripts detected per category is given in parenthesis, next to its classification. Three types of small RNAs were detected: micro RNAs (miRNAs), small nuclear RNAs (snRNAs), and small nucleolar RNAs (snoRNAs). Misc_RNA stands for miscellaneous types of RNAs.

Testis and epididymis expression data comprised over 21,000 transcripts, with at least 10 counts per million reads in the data. Among expressed transcripts, 20,155 were small non-coding RNAs (miRNAs, snRNAs, and snoRNAs) and protein-coding RNAs (mRNA); for more detail, see Figure 1. Prior to differential expression analysis, transcripts with less than two RPKM in at least three samples were removed. After filtering, we considered 17,221 transcripts for differential expression and subsequent analyses, which investigated the co-expression relationships between protein-coding RNA (mRNA) and small non-coding RNAs (miRNAs and snoRNAs) in testicular and epididymis tissues. We performed differential expression analysis contrasting the three tissues sampled, in pairwise comparisons: HE vs. TE, HE vs. TP, and TE vs. TP. To identify the DE transcripts (mRNAs and small RNAs), we used the Limma package in R (Ritchie et al., 2015) to compute the moderated *t*-statistics, using the empirical Bayes methods (eBayes) and the default parameters. The DE transcripts with adjusted *P* value ≤ 0.05 (Benjamini and Hochberg, 1995) and fold change ≥ 2 were considered significant. We generated three lists of DE transcripts, one for each pairwise comparison: HE/TE, HE/TP, and TE/TP.

### Functional Enrichment Analysis of DE Transcripts

The three lists of DE transcripts were the target lists for functional enrichment analyses. We performed the enrichment analysis using the ClueGO v. 2.5.1 bioinformatics tool (Bindea et al., 2009), a plug-in of the Cytoscape software (Shannon et al., 2003). The background gene list for functional enrichment was based on the *Bos taurus* genome, available as a default database in ClueGO. In this analysis, we identified the gene ontology terms (GO terms) and pathways [from the Kyoto Encyclopedia of Genes and Genomes (KEGG) database] that were over-represented in the target DE list. Redundant GO terms were clustered, considering a Kappa score = 0.4, and adjusted *P* values ≤ 0.05 (Bonferroni step-down method) were observed when reporting on significant GO terms or pathways. To improve the functional annotation of the DE transcripts, we cross-checked these lists with the manually curated database for bovine transcription factors (TF) (de Souza et al., 2018).

### miRNA Target Genes Prediction

We predicted the target genes for the DE miRNA using the TargetScan function of R package hoardeR^5^. This function uses all the information stored in the database targetscan.org (release 7.2) that is available for the *Bos taurus* genome in terms of miRNA data. TargetScan predicts the targets of miRNAs by searching for the presence of conserved 8mer, 7mer, and 6mer sites that match the seed region of each miRNA (Lewis et al., 2005). Release 7 of TargetScan uses an improved method to predict targeting efficacy (the context + + model) (Agarwal et al., 2015), uses 3′ UTR profiles that indicate the fraction of mRNA containing each site (Nam et al., 2014), and uses updated miRNA families curated by Chiang et al. (2010) and Fromm et al. (2015). Of note, TargetScan is limited to known sites and 3′ UTR profiles and so it cannot predict all possible interactions between miRNA and target genes.

### Co-expression Network Analysis

We performed a co-expression analysis with the log-normalized expression values of all transcripts (mRNAs and small RNAs that were DE) using the partial correlation and information theory (PCIT) algorithm (Reverter and Chan, 2008). Among the significant correlations according to PCIT, we prioritized the most extreme correlations (higher than 0.95 or lower than -0.95) as stronger evidence of interaction between transcripts. These significant and extreme correlations were used to construct a co-expression network, visualized with the Cytoscape software (Shannon et al., 2003). In the network, we marked as attributes the small RNAs (miRNAs, snoRNA, and snRNA), transcription factors (TFs), the tissue comparison in which the transcript was DE, and the small RNAs presenting significant regulatory impact factor (RIF) values for at least one tissue comparison (Reverter et al., 2010). Also, we pointed out hub transcripts in the network. Hubs are transcripts with higher than the average significant correlations, beyond two standard deviations (i.e., hubs are hyper-connected). In the same context, hub centrality elements are transcripts in the network with higher betweenness centrality than the average (more than two standard deviations), meaning that they tend to link different parts of the network.

### Regulatory Impact Factor Analysis

The regulatory impact of each DE small RNAs over the DE genes for the same comparison analysis was estimated with the RIF algorithm (Reverter et al., 2010). The original application of the RIF algorithm was to determine the regulatory impact of TFs over selected genes (targets) related to a given trait through their expression values between contrasting groups (Reverter et al., 2010). In our experiment, for each pairwise comparison between sampled tissues, we used the RIF algorithm to determine the regulatory impact of each DE small RNA over the DE genes that were identified in the same pairwise comparison. For example, DE small RNA in the TP/HE comparison were tested as potential regulators of the DE genes identified in the TP/TE analyses. The RIF algorithm was selected for this analyses as its predicted regulatory roles have been showcased and validated in previous studies (Bottje et al., 2017; Nolte et al., 2019). A limitation of our analyses is that *in vitro* validations for the predicted co-expression and regulatory relationships were beyond the project scope. To mitigate this limitation, we used RIF in combination with PCIT, TargetScan, and *in silico* analyses of the minimum free energy of miRNA-target hybridization.

### Minimum Free Energy: miRNA and Target Hybridization

The miRNA that were DE, significant according to RIF, and had potential targets with negative co-expression correlations were hypothesized as down-regulators of their targets. When the hypothesis was supported by the identification of binding sites confirmed with TargetScan, the miRNA were subjected to a final analysis: we estimated the minimum free energy (mfe) of the hybridization between the selected miRNA and their confirmed target genes, using RNAhybrid tool (Rehmsmeier et al., 2004). For this, we retrieved the miRNA mature sequence from the miRBase sequence database^6^ and the cDNA sequences of the genes from BioMart (Durinck et al., 2009). A transcript with a mfe less than -20 kcal/mol can be considered a potential target for the miRNA in question (Yen et al., 2019).

## Results

Samples from the head and tail epididymis (HE and TE) and the testicular parenchyma (TP) of *Bos indicus* bulls were used for RNA sequencing. A total of 3,826 DE transcripts (mRNAs and small RNAs) were identified across the tissues in three pairwise comparisons: HE/TE, HE/TP, and TE/TP. A co-expression network was predicted and analyzed, with emphasis on investigating potential regulators of DE genes in these tissues. The network was enriched for male gamete generation and so we infer that the potential regulators of the identified DE genes might contribute to spermatogenesis.

### Transcript Expression Patterns in Male Reproductive Tissues

Reads from RNA-sequencing of HE, TE, and TP were mapped to the genome, and the expression data was summarized per transcript category (Figure 1). All samples considered, the RNA sequencing data comprised of 85.0% mRNAs (17,899) and 10.7% small RNAs, including 812 miRNAs, 746 snRNAs, and 698 snoRNAs. In the bovine reference genome, approx. 13% of all transcripts are small RNAs, and so this is not too far from the 10% identified here. Ribosomal RNA (rRNA) were not well represented as expected in view of the library preparation methods. The library preparation allowed quantifying the expression of mRNAs and small RNAs, but it is also a limitation of this study since it did not enrich for small RNAs and no discovery of small RNAs was conducted. Mitochondrial RNA is not included in Figure 1 because they were less than 1% of the distribution. After the quality control, we kept 17,221 transcripts expressed that were quantified across tissues for all subsequent analyses. The expression pattern of TP samples was different from the epididymis samples (both HE and TE) according to the principal component analysis (PCA) performed, see Supplementary Figure 1.

### Differentially Expressed Transcripts and Functional Enrichment Analysis

**FIGURE 2:**
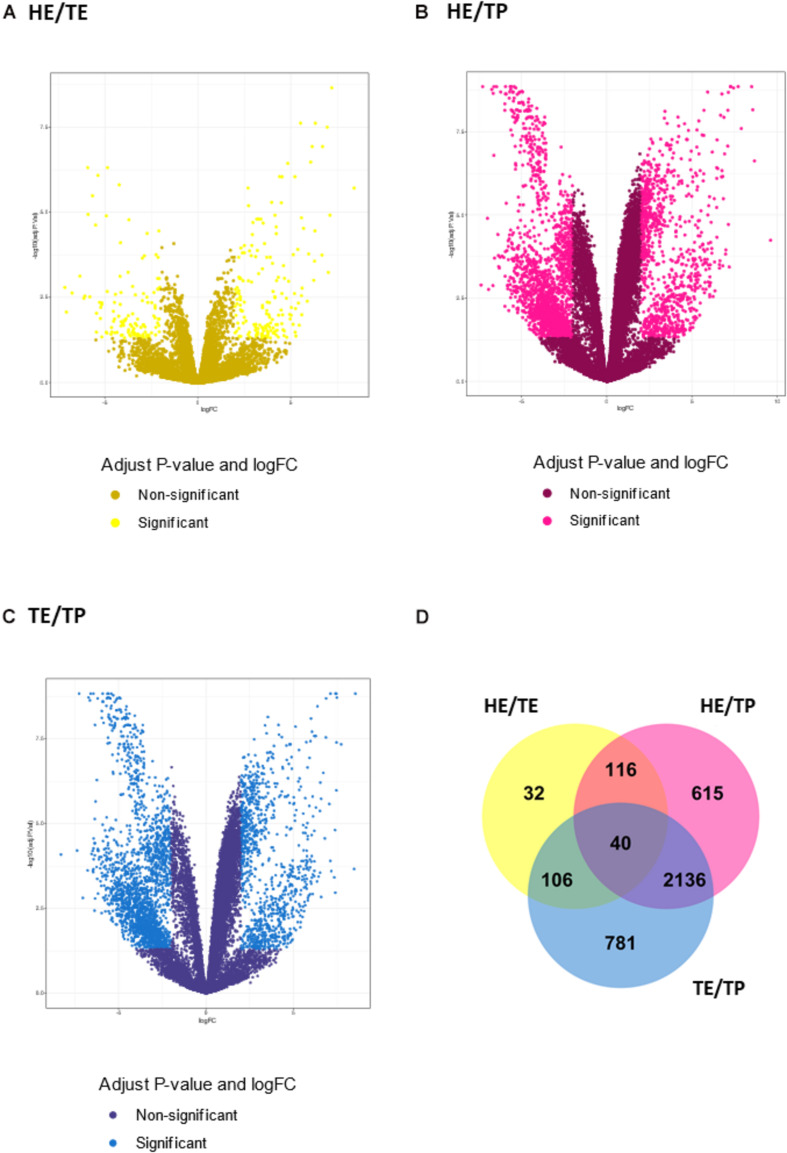
Transcript expression patterns displayed as volcano plots with the log2 fold change in the *x*-axis and the –log10 (*P* value) in the *y*-axis for three pairwise comparisons: **(A)** quantified transcripts in the head versus tail epididymis comparison (HE/TE, in yellow). **(B)** quantified transcripts in the head epididymis versus testicular parenchyma comparison (HE/TP, in pink). **(C)** quantified transcripts in the tail epididymis versus testicular parenchyma comparison (TE/TP, in blue). In **(A–C)**, the light shade dots represent the significantly different genes, while the dark shade dots are not significant. In **(D)**, a Venn diagram summarizes the significantly different transcripts identified in each comparison, including their overlaps.

The number of DE transcripts identified (FDR ≤ 0.05 and log2 fold-change > 2) in each pairwise comparison between HE, TE, and TP are reported in Table 1. The full details on all DE transcripts are provided in Supplementary Table 1. In Supplementary Table 1, positive and negative signals of the log-transformed fold change indicate if the transcript is up- or down-regulated for the first tissue in each comparison (for HE/TE and HE/TP comparisons, a positive fold change represents up-regulation in HE; in TE/TP comparison, a positive fold-change means the transcript was up-regulated in TE). Figure 2 showcases the transcript expression patterns as volcano plots with the fold change plotted against the significance for each transcript, in each of the comparisons. We identified 40 DE transcripts that were in common for all the comparisons (Figure 2D and Supplementary Table 2). Our DE analysis identified a total of 3,826 transcripts that were DE in at least one of the three comparisons.

**TABLE 1:** Summarized differentially expressed (DE) genes and small RNAs in each comparison of male reproductive (epididymis and testis) tissues of *Bos indicus* bulls.

Differentially expressed (DE)	HE/TE^1^	HE/TP^2^	TE/TP^3^	Total
mRNA	268	2,614	2,761	3,442
miRNA	10	108	111	144
snRNA	12	129	127	162
snoRNA	4	56	64	78
**Total**	**294**	**2,907**	**3,063**	**3,826**

*^1^Comparison between HE and TE; ^2^comparison between HE and TP; ^3^comparison between TE and TP. HE, head epididymis; TE, tail epididymis; TP, testicular parenchyma.*

**FIGURE 3:**
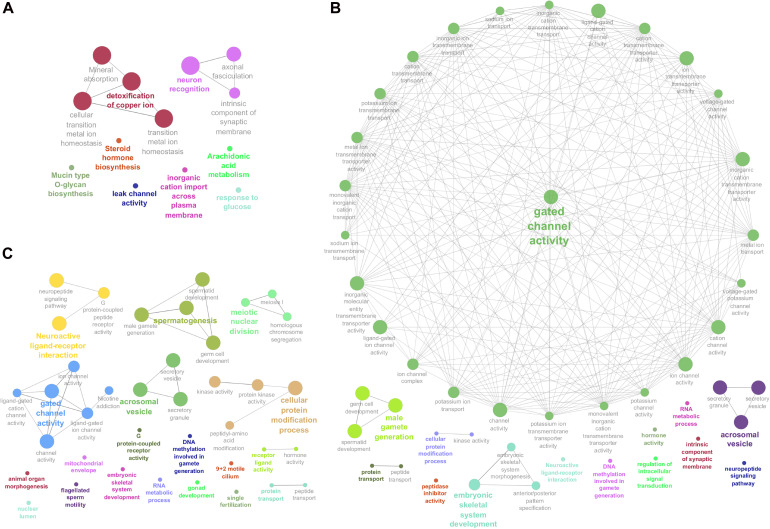
Enriched Genome Ontology (GO) terms and Kyoto Encyclopedia of Genes and Genomes (KEGG) pathways identified by the ClueGO software for each comparison of differentially expressed (DE) analysis between the male reproductive tissues (HE/TE: head vs. tail; HE/TP: head vs. testicular parenchyma; TE/TP: tail vs. testicular parenchyma) of *Bos indicus* bulls. **(A)** Enriched GO terms and KEGG pathways of HE/TE comparison. **(B)** Enriched GO terms and KEGG pathways of HE/TP comparison. **(C)** Enriched GO terms and KEGG pathways of TE/TP comparison. GO terms and KEGG pathways are represented by circles.

Enriched GO terms and KEGG pathways for the total of 3,826 DE transcripts are shown in Figure 3 (details in Supplementary Table 3). The DE genes identified between HE and TE formed a target list that was enriched for nine GO terms and four KEGG pathways. The most significant GO term in the HE/TE comparison was *detoxification of copper ion* (corrected *P* = 2.62 × 10^–7^). DE genes identified between HE and TP were enriched for 46 GO terms and one KEGG pathway. In the third comparison, TE/TP, the DE genes were enriched for 36 GO terms and two KEGG pathways. When TP was compared to the epididymis regions, some of the most significant GO terms were *gated channel activity*, *cellular protein modification*, *male gamete generation*, *neuroactive ligand-receptor interaction*, *spermatogenesis*, and *acrosomal vesicle* (Supplementary Table 3).

### Small RNAs With Regulatory Potential and Co-expression Networks

**FIGURE 4:**
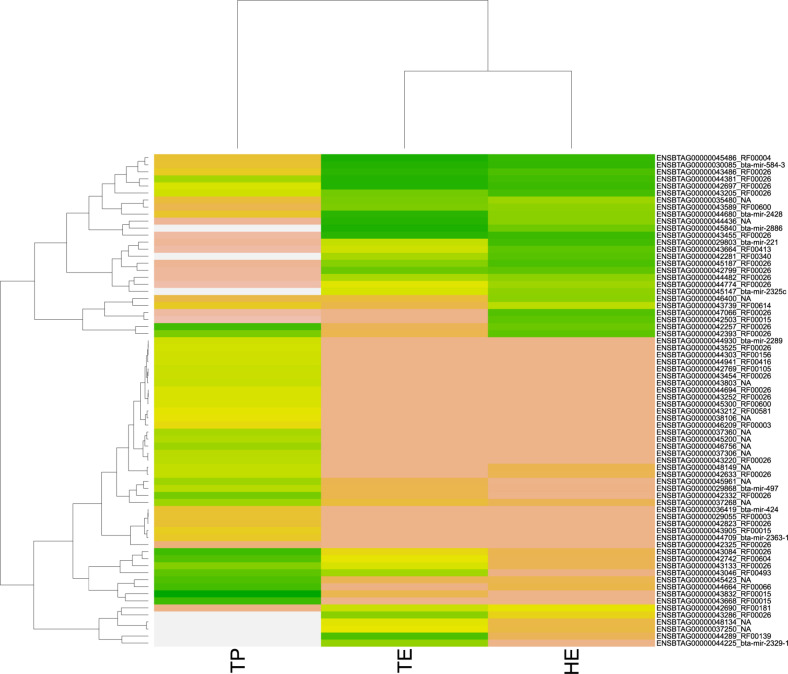
Heatmap of pattern gene expression of small RNAs (snRNAs and miRNAs) with regulatory potential, according to significant values in the regulatory impact factor (RIF) metric. Small RNAs are shown on the *Y*-axis and the total of expression per biopsies of epididymis head (HE), epididymis tail (TE), and testicular parenchyma (TP). The colors correspond to the intensity of expression per tissue. The intensity is scaled by the colors white, yellow, and green. The low expression is represented by white, followed by salmon, yellow, and green (high expression).

Among the 3,826 DE transcripts, 384 were small RNAs and 3,442 were mRNA genes. We identified 71 small RNAs that might modulate the DE genes, according to the significant RIF score (RIF 1 or 2 higher than | 1.96|; Supplementary Table 4). The expression pattern of these 71 small RNAs with regulatory potential differed between samples, across the male reproductive tract (Figure 4). Overall, we observed that small RNAs showed an expression pattern in the testis that was different from their epididymis expression. The difference between the head and tail epididymis was less pronounced, and this is similar to the PCA results for all transcripts.

**FIGURE 5:**
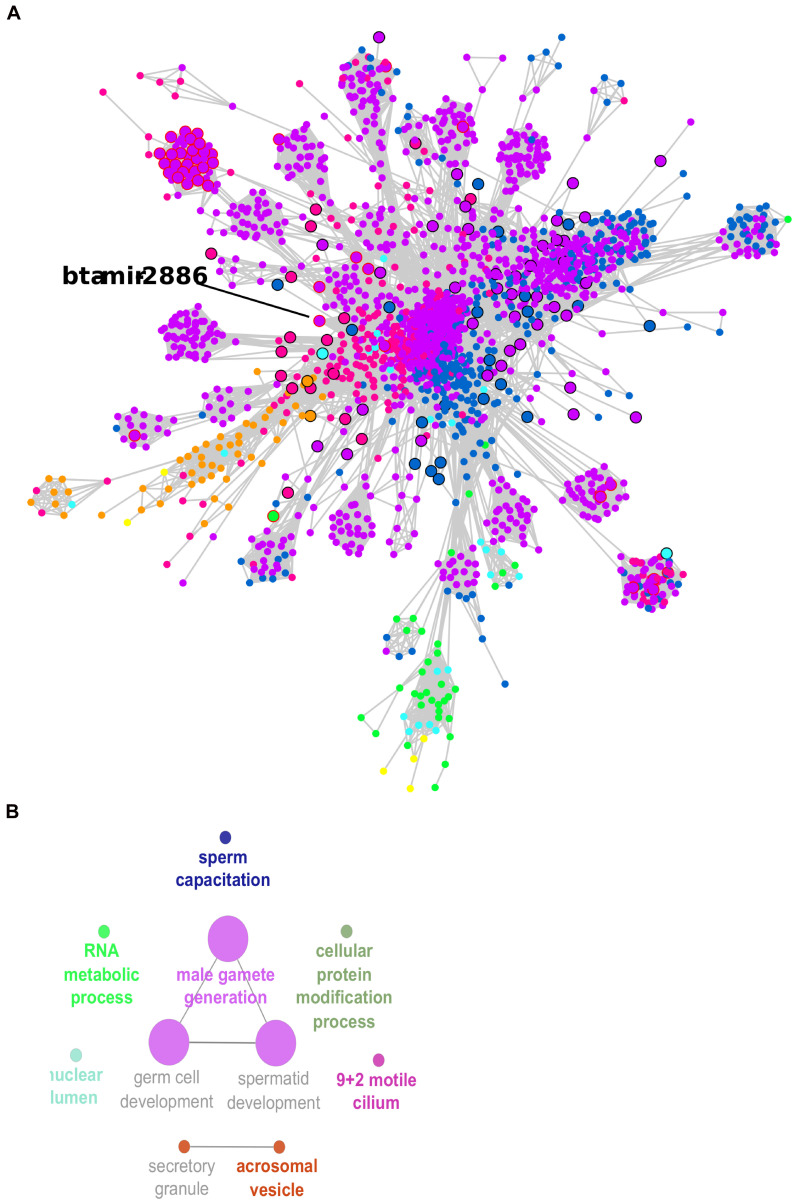
Co-expression network large cluster of differentially expressed (DE) transcripts enriched for male gamete generation. **(A)** Node sizes are proportional to the number of connections (highly connected nodes appear bigger). The nodes are the DE transcripts and the edges are the significant correlations (partial correlation and information theory, PCIT > | 0.95|). Black borders represent hub transcripts. Red borders represent small RNAs with significant regulatory impactor factor values. Node color: yellow for DE only in the head and tail epididymis comparison (HE/TE); pink for DE only in the head epididymis and testicular parenchyma comparison (HE/TP); blue for DE only in the tail epididymis and testicular parenchyma comparison (HE/TP); orange for DE in the HE/TE and the HE/TP comparisons; green for DE in the HE/TE and the TE/TP comparisons; purple for DE in the HE/TP and the TE/TP comparisons; and turquoise for DE in all comparisons. **(B)** Enriched GO terms and KEGG pathways for the cluster represented in **(A)**.

The co-expression network was inferred using significant correlations (> |0.95|). This meant that 3,639 transcripts were nodes linked by 175,052 edges in the network, which is available as a Cytoscape file (Supplementary Material, cys file). The co-expression network was formed by multiple clusters, not all connected to each other (Supplementary Figures 2, 3). A larger cluster with 2,216 transcripts connected through 158,807 edges was the prominent feature in the network. This large cluster was functionally enriched for *male gamete generation*, *germ cell development*, and *sperm capacitation*, among other GO terms (Figure 5 and Supplementary Table 5).

In the large cluster of the co-expression network, the RIF significant small RNA bta-mir-2886 was the hub. Significant correlations suggested 601 co-expressed transcripts for bta-mir-2886, including genes and other small RNA. This is the highest number of connections for a RIF regulator in the network (Supplementary Table 4). Considering the connections between these 601 potential targets and their first neighbors, we observed a total of 1,035 transcripts that were directly or indirectly linked to bta-mir-2886 in the network (Supplementary Table 6). Among the 601 directly co-expressed transcripts, we identified one isoform of U4 spliceosomal RNA with significant RIF value and 38 transcription factors (TF).

Considering the first neighbors of bta-mir-2886 in the network, we identified a total of 241 negative correlations and 360 positive correlations. Among the negative correlations, 204 were mRNA genes. Only three of these potential targets were confirmed to have a site for hybridization with bta-mir-2886 according to TargetScan. The confirmed targets were *ELOVL3*, *FEZF2*, and *HOXA13*. All three had a mfe that is further evidence for bta-mir-2886 acting as their down-regulator: −28.5 kcal/mol for *ELOVL3*, -34.95 kcal/mol for *FEZF2*, and -35.75 kcal/mol for *HOXA13*. All TargetScan results for bta-mir-2886 are provided in Supplementary Table 7. TargetScan analyses of all DE miRNAs were performed and provided evidence for 1,846 DE genes that can be proposed as targets of miRNA regulation in the male reproductive tract. However, TargetScan analyses could not explain all the co-expression observed between miRNA and genes. This result is expected, since co-expression is not necessarily caused by direct hybridization and regulation, as there are many – and complex – molecular mechanisms that can lead to co-expression (Fionda, 2019).

## Discussion

### Transcript Expression Patterns in Male Reproductive Tissues

In this study, we identified 17,221 transcripts quantified in bovine samples of the head and tail epididymis and the testicular parenchyma (HE, TE, and TP). This amounts to 64% of the genome (or 17,221 of 26,740 transcripts). The expression of a relatively large number of genes and small RNAs confirmed previous reports that suggest the testis as a good sample for functional genome annotation. In humans, the testis expressed a larger number of genes in comparison to other tissues (Uhlén et al., 2015). Harhay et al. (2010) has shown a cluster of genes exclusively expressed in the testis of bovine. The data we reported on is available through the Functional Annotation of Animal Genomes (FAANG) Consortium for future reference and further use (see text footnote 1). Herein, we focus on differential expression analyses, functional enrichment, co-expression network analyses, and regulatory impact metrics (RIF and additional *in silico* tests) that point to potential modulators of transcription in male reproductive tissues.

We identified 71 small RNAs with significant RIF values, interpreted as potential contributors to the modulation of over 3,000 DE transcripts. The expression patterns of these 71 potential regulators were similar to the overall pattern observed, with the testis expression contrasting with the epididymis expression. The expression patterns in all epididymis samples were relatively similar. These expression patterns might reflect the specific function and distinguished cell populations of the studied samples. In boar samples, Guyonnet et al. (2009) has observed different patterns of gene expression in the testis and epididymis. A different role for small regulatory RNAs has been proposed, specific for each region of the male reproductive system, associated with regional function. For example, Guyonnet et al. (2009) has found that genes related to spermatogenesis are more prominent in the testis, compared to the epididymis. Their observation corroborates our findings. In the HE/TE comparison, only two of the 71 small RNAs detected with RIF were DE and relatively fewer genes were DE. Still, the epididymis regions have different roles in the biological processes involved in sperm maturation and transit, and this has been linked to the regionalization of gene expression patterns (Guyonnet et al., 2009; Belleannée et al., 2012; Browne et al., 2015). Our results indicated that specific small RNAs might play regulatory roles that contribute to the regionalization of gene expression in the reproductive system of *Bos indicus* bulls.

### *Detoxification of Copper Ion* in the Head Epididymis

The regionalization of gene expression in the reproductive system can be discussed in light of the enriched GO terms and pathways associated with the DE transcripts. The most significant GO term in the HE/TE comparison was *detoxification of copper ion*. Genes associated with *detoxification of copper ion* were up-regulated in the head, including three members of the metallothionein family: *MT1A*, *MT2A*, and *MT1E* (Wong et al., 2017). These genes are involved in metal homeostasis and metal detoxification (Schulkens et al., 2014) and can protect cells from oxidative stress (Sutherland and Stillman, 2011). The expression of *MT1A*, *MT2A*, and *MT1E* is up-regulated in the presence of copper in adult human prostatic cell lines (Cu treatment vs. untreated) (Bigagli et al., 2010). In dogs with hepatitis, *MT1A* and *MT2A* expression levels decrease together with a copper concentration in hepatic cells (Dirksen et al., 2017). Protection from oxidative stress is important for sperm cells; in fact, the molecular environment of the epididymis is crucial for sperm maturation and capacitation (Belleannée et al., 2012). Increased dietary copper is associated to improved spermatozoa mobility and quality in bulls (Hidiroglou, 1979). However, high levels of copper can affect cell homeostasis and be detrimental to sperm quality and its fertilization capacity (Roblero, 1996). High levels of copper can disturb the integrity of the epididymis and affect sperm maturation (Xu et al., 1985). In this context, our results point to *MT1A*, *MT2A*, and *MT1E* as genes that may assist with copper homeostasis in the head epididymis and might have a role in sperm maturation.

The regionalization of gene expression in the male reproductive system is likely a consequence of regulatory mechanisms, including small RNAs that target genes post-transcriptionally. Bta-mir-362 had a negative co-expression correlation with *MT1E* and *MT1A* (lower than −0.95) and might down-regulate these genes. Bta-mir-362 is reported to contribute to spermatogenesis processes in pigs too (Ran et al., 2018). In short, this miRNA might modulate genes involved with *detoxification of copper ion* in the epididymis, and as a consequence, it might affect sperm quality.

### *Gated Channel Activity*, Ions, and Water Transport

In our study, the GO term *gated channel activity* and other terms related to ion transport and channel activity were significant for the comparisons between the epididymis and testis. In both the HE/TP and the TE/TP comparisons, DE genes suggest that ion channels are relevant to spermatogenesis. This result is in agreement with previous knowledge, because ions such as Ca^2+^ and Na^+^ contribute to the acrosomal reaction, hyper-activation, sperm capacitation, and sperm quality (Mirnamniha et al., 2019).

Differentially expressed and enrichment analyses suggest regionalization of expression patterns in the male reproductive system for genes that code proteins related to ion channels. Specific proteins related to ion channels and solute transporters are responsible for the epididymis homeostasis and for the luminal environment that is adequate to sperm maturation (Belleannée et al., 2012). In short, ion channels, anions, cations, and water transport molecules (i.e., AQPs) are involved in the control of the luminal fluid (Browne et al., 2015).

The DE genes *ATP6V0A4*, *ATP6V0D2*, *ATP6V1G3*, *CFTR*, *SCNN1G*, and *SCNN4A* are related to ion channel activity and were up-regulated in the epididymis when compared to the testis. The *ATP6V0A4*, *ATP6V0D2*, and *ATP6V1G3* genes were up-regulated in both HE/TP and TE/TP comparisons. They code for proteins that compose the subunit of vacuolar H + –ATPase (V-ATPase) (Wagner et al., 2004). The V-ATPase is a multi-subunit ATP-driven proton pump (Pamarthy et al., 2018), with influence in the acidification of luminal fluid (Brown et al., 1992; Roy et al., 2013) that may help sperm maturation (Pamarthy et al., 2018). Failure in luminal fluid acidification can result in poor sperm maturation and infertility (Breton et al., 1996). In this context, a higher expression of these genes in the epididymis suggests they are relevant to sperm maturation in *Bos indicus* bulls.

The cystic fibrosis transmembrane conductance regulator (*CFTR*) gene was up-regulated in the head epididymis when compared to the testis. The CFTR channel is involved in sperm capacitation (Touré, 2019) and can contribute to the Cl^–^ and bicarbonate fluxes (Touré, 2019). The DE gene *SLC26A3*, up-regulated in the epididymis, is essential to bicarbonate fluxes and interacts with the CFTR channel (Touré, 2019). The *SLC26A*3 knockout mice present lesions in the epididymis and sperm reduction (El Khouri et al., 2018). CFTR and epithelial Na^+^ channel (ENaC) contribute to sperm capacitation (Visconti et al., 2011; Sharma and Hanukoglu, 2019). ENaC is probably involved in the uptake of Na^+^ ions from the epididymal lumen into the cells. Like CFTR channels, ENaC channels are observed in patterns along the length of the mouse and rat epididymis (Sharma and Hanukoglu, 2019). Two genes that code for ENaC proteins, *SCNN1G* and *SCNN4A*, were up-regulated in the epididymis when compared to the testis. *SCNN1G* was up-regulated in the head (HE/TP) while *SCNN4A* was up-regulated in both the head (HE/TP) and tail (TE/TP). The regional regulation of genes coding for ENaC proteins might be an evidence of their role in sperm maturation. The bovine DE patterns discussed here conform to the expectations from studies in other species and might reveal mechanisms that are relevant to male fertility across mammals.

Another gene that was up-regulated in the head and tail epididymis when compared to the testis was *AQP9*, an aquaporin. Aquaporins (AQPs) are channels of proteins that facilitate the movement of water across the plasma membrane and contribute to epididymal sperm concentration (Belleannée et al., 2012; Schimming et al., 2017). *AQP9* has been previously reported as expressed in the epididymis and related to water resorption in the proximal epididymis (Belleannée et al., 2012). Gene expression patterns of AQPs have been related to disorders of the male reproductive system in mammals (Huang et al., 2006). Therefore, we hypothesize that *AQP9* might affect water transport in the epididymis in *Bos indicus*.

Small RNAs might modulate DE genes involved in *gated channel activity*, ion channels, and water transport. We predicted interactions between 24 small RNAs (15 miRNAs and 9 snRNA) and the DE genes discussed above. For example, bta-mir-2886 was co-expressed with *AQP9* and *CFTR*. The snRNA RF00026 (U6 spliceosomal RNA) was co-expressed with *ATP6V0D2*, *AQP9*, *CFTR*, and *SLC26A3*. It is possible then that bta-mir-2886 and RF00026 among other small RNAs modulate *gated channel activity*, ions, and water transport, affecting epididymis function.

### Neuroactive Ligand-Receptors and Spermatogenesis

Among the DE transcripts, we identified genes related to prolactin, GABA, and muscarinic acetylcholine receptors, all part of the enriched *neuroactive ligand-receptor interaction* pathway. Overall, this pathway seems more important to testicular function than to the epididymis, with a few exceptions as discussed below.

Prolactin is a peptide hormone that acts via its transmembrane receptor (Raut et al., 2019). In our study, nine DE genes related prolactin signaling – *PRL*, *PRLH*, *PRP1*, *PRP14*, *PRP2*, *PRP4*, *PRP6*, *PRP8*, and *PRP9 –* were up-regulated in the testis when compared to the epididymis. Prolactin receptors are expressed in the testis of humans (Jabbour and Kelly, 1997) and bulls (Pratt et al., 2015). Prolactin signaling affects the male reproduction system (Hermanns and Hafez, 1981) as it interferers with steroidogenesis and spermatogenesis (Jabbour and Kelly, 1997). Also, prolactin signaling relates to testosterone concentration (Franchimont, 1983). In summary, the DE analyses recapitulated some known biology of testicular function and suggested prolactin genes that were regulated in male tissues.

GABA receptors were previously reported in the testis and sperm of mice (He et al., 2001, 2003). We identified 10 GABA receptors as DE genes in the HE/TP and TE/TP comparisons: *GABBR2*, *GABRA2*, *GABRA3*, *GABRA4*, *GABRA5*, *GABRB1*, *GABRB3*, *GABRG2*, *GABRP*, and *GABRQ*. Three receptors (*GABRG2*, *GABRB1*, and *GABRA5*) had higher expression in the epididymis, while the other seven were up-regulated in the testis. In the testis, GABA receptors affect the Leydig cell function, influence germ cell maturation (Geigerseder et al., 2003), and regulate spermatogenesis (Geigerseder et al., 2003; Kanbara et al., 2005; Du et al., 2013). The function of these DE GABA receptors, with expression that is specific to each region of the male system examined, requires further research.

Three DE genes, *CHRM1*, *CHRM2*, and *CHRM3*, are receptors related to muscarinic acetylcholine signaling. Muscarinic acetylcholine receptors (or MAChRs) are part of the regulatory mechanisms in the male reproductive system (Borges et al., 2001; Avellar et al., 2010). MAChRs regulate testicular cell function (Borges et al., 2001) and can influence the luminal fluid composition (Avellar et al., 2010). In our study, *CHRM1*, *CHRM2*, and *CHRM3* were up-regulated in the testis compared to the epididymis, and so we suggested that MAChRs might contribute to testicular function in *Bos indicus* bulls.

Among DE genes of the *neuroactive ligand-receptor interaction* pathway, 10 were connected to the larger network cluster and directly or indirectly linked to bta-mir-2886. The prolactin signaling genes *PR2*, *PRP14*, and *PRP9* were predicted to interact directly with bta-mir-2886, while *GABRA5* was a second neighbor of this same miRNA. In short, it is possible that bta-mir-2886 and other small RNAs regulate genes in the *neuroactive ligand-receptor interaction* pathway that might affect spermatogenesis.

### Co-expression Network, Small RNAs, and Male Gamete Generation

Differentially expressed transcripts associated with the GO term *male gamete generation* were enriched in the comparisons between the epididymis and testis, being the third most significant term in both HE/TP and TE/TP comparisons. This same GO term was significant for transcripts in the larger cluster of the co-expression network. Four well-known regulators of spermatogenesis were among the DE transcripts associated with *male gamete generation*: *RFX2*, *HORMAD1*, *CCDC36*, and *DAZL*. The gene *RFX2* is an essential transcription factor in the regulation of spermatogenesis (Kistler et al., 2015; Pandey et al., 2019), which is expressed in spermatocytes and spermatids in mice (Pandey et al., 2019). The *RFX2*-deficient mice have completely blocked spermatogenesis (Kistler et al., 2015). *HORMADA1* is key during the meiosis and it possibly interacts with *CCDC36* (Stanzione et al., 2016). *DAZL* stands for “deleted in azoospermia like,” and it codes for a RNA-binding protein that is localized to the nucleus of spermatogonia, but relocates to the cytoplasm during meiosis, where it persists in spermatids and spermatozoa. *DAZL* is highly expressed in the testis of sheep with sexual maturity (Yuan et al., 2020) and may have a role in sex differentiation (Rossitto et al., 2015). All four genes were up-regulated in the testis when compared to the epididymis, which is expected since *male gamete generation* is a characteristic of the testis. Two of these well-known regulators, *RFX2* and *CCDC36*, were also nodes in the larger network cluster.

In the larger network cluster, five out of 10 significant GO terms were very specific to spermatogenesis: *male gamete generation*, *spermatid development*, *germ cell development*, *acrosomal vesicle*, and *sperm capacitation.* Therefore, the small RNAs that were identified as potential regulators of this cluster of highly connected DE genes might be proposed as potential regulators of spermatogenesis. We identified 228 small RNAs in the larger network cluster and 43 of these had significant RIF values; they were 20 snRNAs, 8 snoRNAs, and 15 miRNAs. One of the significant miRNAs was bta-mir-2886, up-regulated in the epididymis for two pairwise comparisons (HE/TP and TE/TP), with a high fold change in both (approx. 7). We propose that bta-mir-2886, through its 601 co-expressed transcripts and additional 434 first neighbors, might affect spermatogenesis in *Bos indicus* bulls.

In our study, most of the correlations between bta-mir-2886 and predicted targets were positive, including for *AQP9* and *CFTR*. We speculated that this miRNA might be indirectly regulating the expression of co-expressed transcripts, with which it presents positive correlations, by inhibiting their negative regulators. This indirect mechanism was suggested previously by Ritchie et al. (2009). We observed 204 predicted negative correlations with bta-mir-2886. Among negative correlations, we identified three genes that may be down-regulated by bta-mir-2886, which were confirmed by TargetScan and had mfe below -20 kcal/mol. They were *ELOVL3*, *FEZF2*, and *HOXA13*.

*HOXA13* was among 23 genes of the Hox family that were DE in our study. Hox family transcription factors are expressed in the male reproductive tract (Lindsey and Wilkinson, 1996), including the human testis (Zhu et al., 2016) and mice epididymis (Bomgardner et al., 2003; Raines et al., 2013). Hox genes act in spermatogenesis and sperm maturation (Lindsey and Wilkinson, 1996). Zhu et al. (2016) have reported Hox genes as regulators of meiosis in the human testis. Mutations in *HOXA13* were associated to male infertility in mice (Post and Innis, 1999). Further studies could investigate the role of *HOXA13* in bull fertility.

## Conclusion

Our results indicate that bta-mir-2886, among other small RNAs, are co-expressed with DE genes that may contribute to spermatogenesis and sperm maturation in the testis and epididymis of *Bos indicus* bulls. Although our study predicts potential regulators of gene expression in the testis and the epididymis of *Bos indicus* bulls, further work is necessary to confirm our findings and detail the roles played by small RNAs in spermatogenesis.

## Data Availability Statement

The datasets presented in this study can be found in online repositories. The names of the repository/repositories and accession number(s) can be found below: https://data.faang.org/organism, SAMEA104495807.

## Ethics Statement

The animal study was reviewed and approved by the Ethics Committee of the University of Queensland, Brisbane, Australia (protocol number: ANRFA/SCMB/094/16).

## Author Contributions

MF conceived the idea of this study. MF, EM, and LP-N performed field and laboratory work. JE prepared and sequenced the RNA libraries. AdL, RP, JA, and AR performed the bioinformatics and data analysis. AdL, JA, and MF drafted the manuscript. All authors contributed to the interpretation of results, discussion, review of the concepts in this manuscript, and agreed to be responsible for the content of this study.

## Conflict of Interest

The authors declare that the research was conducted in the absence of any commercial or financial relationships that could be construed as a potential conflict of interest.

## Footnotes

1 https://data.faang.org/home

2 https://www.bioinformatics.babraham.ac.uk/projects/trim_galore/

3 https://www.bioinformatics.babraham.ac.uk/projects/fastqc/

4 https://www.openagrar.de/servlets/MCRFileNodeServlet/openagrar_derivate_00022208/

5 https://cran.r-project.org/web/packages/hoardeR/index.html

6 http://www.mirbase.org/
