# Supplementary material for: Network Analyses Predict Small RNAs That Might Modulate Gene Expression in the Testis and Epididymis of Bos indicus Bulls
Source: Front Genet. 2021 Apr 30;12:610116. doi: 10.3389/fgene.2021.610116 (PMC8120238; doi:10.3389/fgene.2021.610116)
Supplement: Supplementary file 2 [file Data_Sheet_2.docx]

Supplementary Figures

**Network analyses predicts regulators of gene expression in testis and epididymis of *Bos indicus* bulls**

**
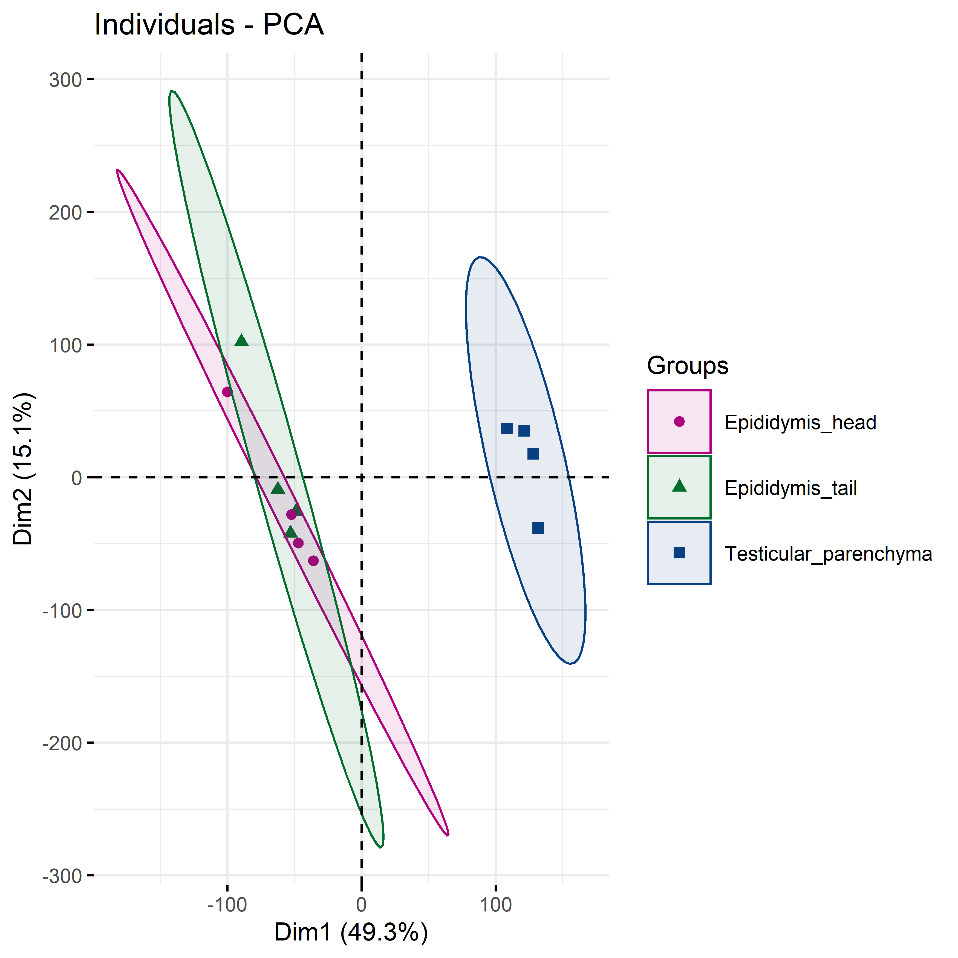
**

**Supplementary Figure S1**. Principal component analysis (PCA) of male reproductive tissues (epididymis and testis): gene expression profiles in *Bos indicus* bulls. After PCA, the figure was created by plotting PC1 (Dim1) against PC2 (Dim2). The testicular parenchyma samples represented as blue squares clustered together, and away from the epididymis samples represented as magenta dots (epididymis head) and green triangles (epididymis tail). Epididymis head and tail expression profiles were similar according to PCA. For each tissue, the expression profile of 4 samples was available and is shown.


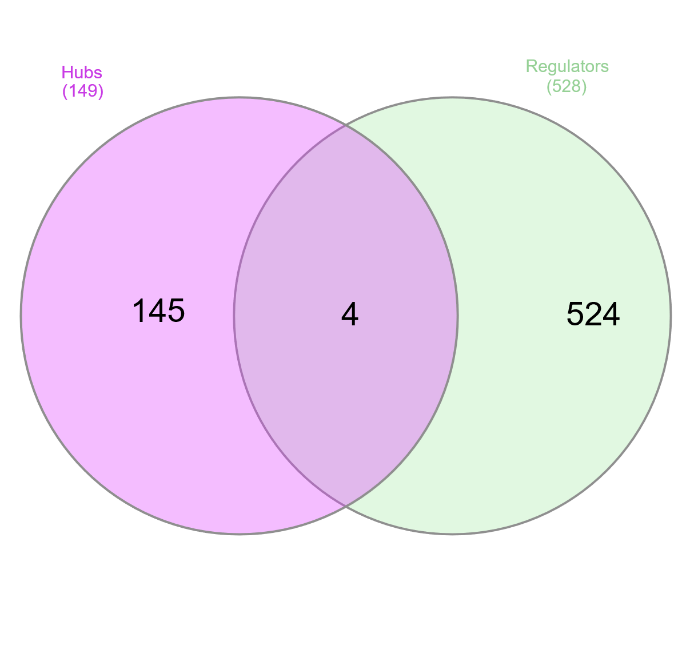


**Supplementary Figure S2**. Venn diagram of the differentially expressed (DE) transcripts that were also either 1) Regulators (small RNAs and transcription factors), or 2) were Hubs in the co-expression network analyses. Among the 528DE transcripts regulators four were also Hubs. Hubs are DE transcripts with a significantly large number of connections in the co-expression network predicted with PCIT analysis (see Figure S3).


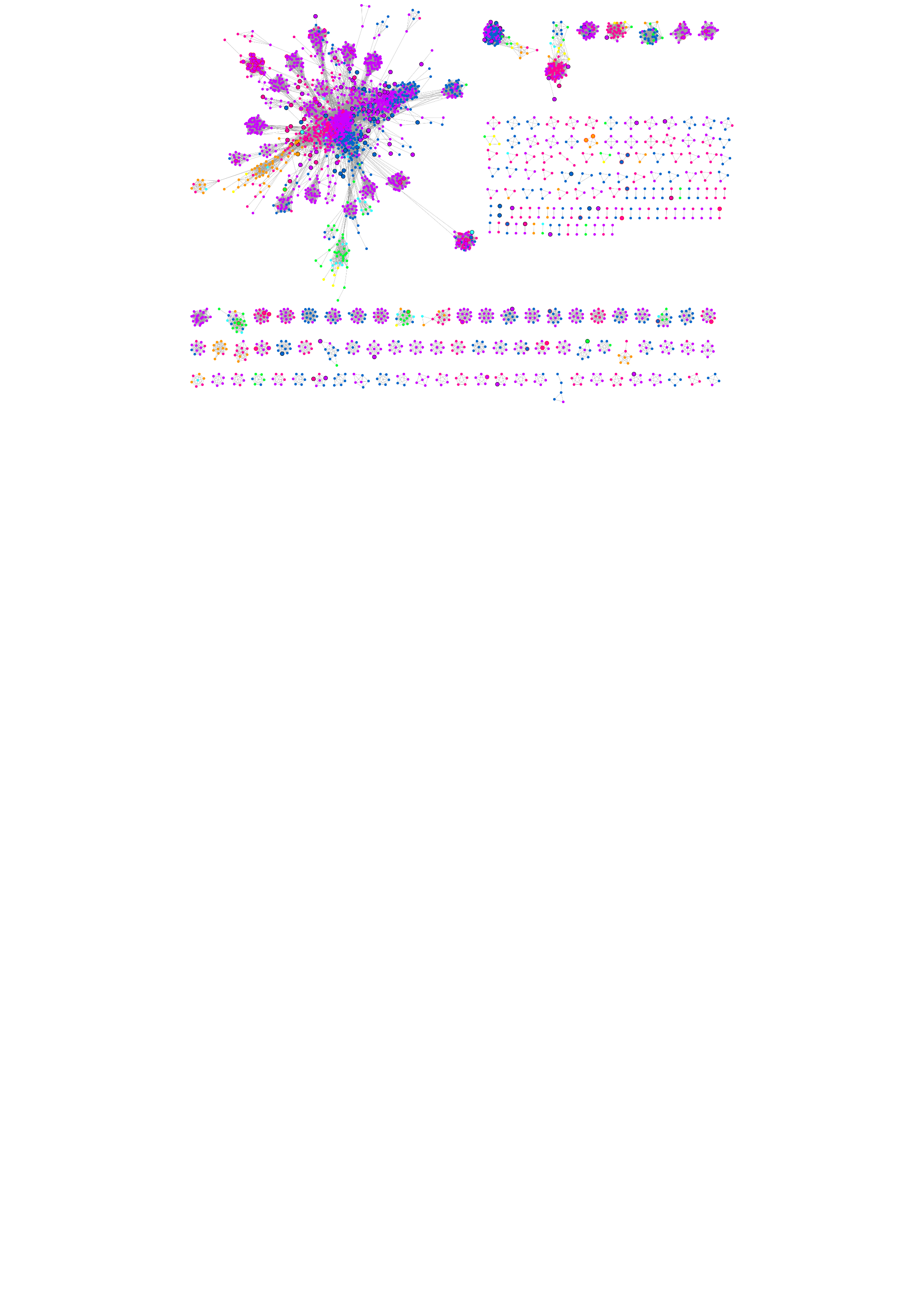


**Figure S3**. Gene co-expression network diagram of differentially expressed (DE) transcripts that connected with regulators (DE small RNAs and DE transcription factors) or Hubs (DE transcripts with a high number of connections). The transcripts in this network were DE in at least one of the pair-wise tissue comparisons, between head epididymis, tail epididymis and testicular parenchyma. The nodes in the diagram are the DE transcripts and the edges are the significant high correlations (PCIT > |0.95|) inferred by PCIT algorithm. Nodes marker with a black border represent Hubs. Nodes with red borders are the small RNAs with significant regulatory impactor factor (RIF) values. Yellow nodes are the transcripts that were DE only in the comparison between head and tail of the epididymis (HE/TE). Pink nodes are transcripts that were DE only in the comparison between head epididymis and testicular parenchyma (HE/TP). Blue nodes are transcripts that were DE only in the comparison between tail epididymis and testicular parenchyma (TE.TP). Orange nodes are transcripts that were DE in both HE/TE and HE/TP comparisons. Green nodes are transcripts that were DE in both HE/TE and TE/TP comparisons. Purple nodes are transcripts that were DE in both HE/TP and TE/TP comparisons. Turquoise nodes are transcripts that were DE in all comparisons.
